# Two distinct mechanisms lead to either oocyte or spermatocyte decrease in *C*. *elegans* after whole developmental exposure to γ-rays

**DOI:** 10.1371/journal.pone.0294766

**Published:** 2023-11-27

**Authors:** Elizabeth Dufourcq Sekatcheff, Christian Godon, Aymeric Bailly, Loïc Quevarec, Virginie Camilleri, Simon Galas, Sandrine Frelon

**Affiliations:** 1 Institut de Radioprotection et de Sûreté Nucléaire (IRSN), PSE-ENV/SRTE/LECO, F-13115, Saint Paul-Lez-Durance, France; 2 Institut de Biosciences et Biotechnologies Aix-Marseille, Aix Marseille University, CEA, CNRS, BIAM, CEA Cadarache, 13108, Saint Paul-Lez-Durance, France; 3 CRBM, CNRS, Université de Montpellier, UMR5237, Montpellier, 34090, France; 4 CNRS, ENSCM, IBMM Université de Montpellier, 34093, Montpellier, France; East Carolina University, UNITED STATES

## Abstract

Wildlife is subject to various sources of pollution, including ionizing radiation. Adverse effects can impact the survival, growth, or reproduction of organisms, later affecting population dynamics. In invertebrates, reproduction, which directly impacts population dynamics, has been found to be the most radiosensitive endpoint. Understanding the underlying molecular pathways inducing this reproduction decrease can help to comprehend species-specific differences in radiosensitivity. From our previous studies, we found that decrease in reproduction is life stage dependent in the roundworm *Caenorhabditis elegans*, possibly resulting from an accumulation of damages during germ cell development and gamete differentiation. To go further, we used the same experimental design to assess more precisely the molecular determinants of reproductive toxicity, primarily decreases in gamete number. As before, worms were chronically exposed to 50 mGy·h^−1^ external gamma ionizing radiation throughout different developmental periods (namely embryogenesis, gametogenesis, and full development). To enable cross species extrapolation, conserved molecular pathways across invertebrates and vertebrates were analysed: apoptosis and MAP kinase Ras/ERK (MPK-1), both involved in reproduction and stress responses. Our results showed that these pathways are life-stage dependent, resulting from an accumulation of damages upon chronic exposure to IR throughout the life development. The Ras/ERK pathway was activated in our conditions in the pachytene region of the gonad where it regulates cell fate including apoptosis, but not in the ovulation zone, where it controls oocyte maturation and ovulation. Additionally, assessment of germ cell proliferation via Ras/ERK pathway showed no effect. Finally, a functional analysis of apoptosis revealed that while the decrease of the ovulation rate is caused by DNA-damaged induced apoptosis, this process does not occur in spermatocytes. Thus, sperm decrease seems to be mediated via another mechanism, probably a decrease in germ cell proliferation speed that needs further investigation to better characterize sex-specific responses to IR exposure. These results are of main importance to describe radio-induced reprotoxic effects and contribute as weight of evidence for the AOP #396 “Deposition of ionizing energy leads to population decline via impaired meiosis”.

## Introduction

Because of the increased number of pollutants that accumulate in the environment every year, there is a growing need to expedite and enhance their screening for toxicity. Risk assessment for ecosystems *per se* is a growing concern, especially since the Convention on Biological Diversity of 1992, all the more in the one health concept cross-connecting the health of animals, ecosystems and humans [1]. This assessment requires both a robust knowledge of the relationship between environmental exposure and induced adverse effects in order to propose reference levels, and a good description and structuration of the data from the first biological events to the observed adverse outcomes in order to characterize the mode of action of different pollutants. To do so, Adverse Outcome Pathways (AOPs), were proposed by the OECD a few years ago as a conceptual framework that enables the structuration of the data and *in fine* a classification of pollutants according to the biological pathways they can trigger [2]. In this context, data acquisition and structure on chronic exposure to ionizing radiation (IR), naturally occurring but also reinforced due to anthropic activities, are of main interest. Indeed, IR share common early biological responses, namely oxidative stress induction via reactive oxygen species (ROS) production [3,4] or direct ionization of molecules (DNA, lipids, proteins, etc.), with numerous chemicals.

These primary processes can lead to adverse effects on larger scales i.e., individual and population via different pathways. In particular, reproduction, which directly impacts population dynamics, is the most radiosensitive individual parameter in many species, including invertebrates and more precisely *Caenorhabditis elegans* [5–9]. However, the early molecular events of this reproduction decrease are still not all elucidated. From our previous studies, we showed that *C*. *elegans* chronic exposure to gamma rays at 50 mGy.h^-1^ (LOAEL for *C*. *elegans* reproduction endpoint after chronic exposure of one generation), lead to significant brood size decrease. This decrease was found to be concomitant with sperm number and ovulation rate decrease. However, no effect on hatching success was observed, suggesting no embryonic damage at this dose rate [10–12]. We concluded that ~90% of the individual brood size decrease can be attributed to sperm decrease, which appears to be compensated by the increase of male spawning in the population [13]. The remaining ~10% brood size decrease can be attributed to other, yet unexplained mechanisms probably linked to the ovulation process [11]. From these studies, the AOP #396 “Deposition of ionizing energy leads to population decline via impaired meiosis” was developed and published in AOP wiki. Some key events linked to germline development and gamete decrease that remained to be investigated were further assessed in the present study.

Chronic exposure to IR triggers specific pathways in the germline such as DNA damage, apoptosis, cell cycle, stress response pathways (DAF-16/FOXO) and gamete proliferation pathways [10,14,15]. Among those, the Ras/ERK (MPK-1) pathway is of particular interest because it is at the crossroads of observed effects. It acts to control several developmental processes in *C*. *elegans* hermaphrodite germline, including the regulation of spermatogenesis/oogenesis transition [16,17], and thus sperm count [18,19] and is also involved in stress response pathways, especially after irradiation [20,21]. In *C*. *elegans*, MPK-1 expression is spatially and temporally dynamic, making it possible to distinguish the various biological processes it regulates according to its zone of expression in the gonad and at different developmental stages [22]. Notably, in the pachytene zone, MPK-1 controls the cell progression into meiosis (transition from distal to proximal pachytene) and the spermatogenesis/oogenesis transition, while in developing oocytes, it controls oocyte maturation and ovulation.

A more common stress response pathway is the apoptotic pathway which was observed after both chronic and acute exposure to gamma rays in *C*. *elegans* [12,15,23]. However, its direct link with brood size decrease remains to be demonstrated. While in *C*. *elegans* prior studies have shown that, in male germ cells, neither DNA-damage induced nor physiological apoptosis occurs upon acute exposure to IR [24–26], male germ cells specific response to chronic exposure remains untreated. Interestingly, in response to DNA damage, apoptotic pathway is triggered by other regulators than physiological apoptosis (i.e., normal apoptosis occurring during oogenesis) [26] but the downstream core apoptotic pathway remains the same, i.e. activation of the protease CED-3 caspase that cleaves specific substrates inducing killing and phagocytosis of the cell [27]. Thus, we hypothesize that if sperm decrease is caused by the apoptotic pathway, direct inhibition of CED-3 should rescue the sperm decrease phenotype. Moreover, *C*. *elegans* germ cells apoptosis is restricted to late pachytene cells at the L4 stage, which makes differential life-stage exposure all the more relevant to elucidate toxic mechanisms [26].

Thus, in order to fill some knowledge gaps in the cascade of events leading to population decline after chronic exposure to ionizing radiation (AOP 396), we investigated the link between brood size decrease and pathways known to be involved in both stress response and developmental processes in the *C*. *elegans* germline, i.e., the Ras/ERK (MPK-1) pathway via dpMPK-1 staining in the germline, germ cell count, and the core apoptotic pathway via CED-3 loss of function mutant. We hypothesize that chronic irradiation during *C*. *elegans* development could induce (1) spermatocytes decrease either via apoptosis (core apoptotic pathway) or precocious transition from spermatogenesis to oogenesis (Ras/ERK pathway), (2) oocyte decrease either via apoptosis (core apoptotic pathway) or ovulation defects (Ras/ERK pathway). To do so, nematodes were chronically irradiated at different life-stages following the same experimental design of our previous study (e.g., embryogenesis, early gonadogenesis, whole development) [11]. In all scenarios brood size, ovulation rate and sperm number were assessed.

## Material and methods

### Strains and maintenance

The following strains were provided by the Caenorhabditis Genetics Centre (University of Minnesota, St. Paul, MN, USA): the Bristol N2 strain was used as a wild type, the MT1522 (*ced-3(n717))* strain was used for the analysis of apoptotic pathway. Nematodes were maintained as described previously [11].

### Antibodies and reagents

The following antibodies and reagents were used in this study: Sigma monoclonal anti-activated MAP kinase (diphosphorylated ERK-1&2) antibody (M8159), Donkey anti-Mouse IgG (H+L) Highly Cross-Adsorbed Secondary Antibody, Alexa Fluor Plus 555 (InVitrogen, Waltham, MA, USA), Vectashield antifade mounting medium with DAPI (Vector Laboratories, Inc., Burlingame, CA, USA).

### Irradiation design

Worms were synchronized and irradiated in the Mini Irradiator for Radio Ecology (MIRE) facility at the IRSN laboratory, (Cadarache, France) as previously described (11). Prior to irradiation, a Monte Carlo Simulation was performed to determine the correct position of the petri dish around the 137Cs source to yield a final dose of 50 mGy.h-1 for each replicate. The final dose uptake was measured using RPL (radio photo luminescent) dosimeters on each plate. Wildtype worms (later stained with DAPI and MPK-1 antibody) were irradiated according to three exposure scenarios (S1 Fig; SC1 –in utero until embryogenesis; SC2 –in utero until the beginning of gametogenesis; SC3 –in utero until the end of development). Details about exposure scenarios are given in Dufourcq Sekatcheff et al, 2019 (11) and in S1 Fig. The MT1522 strain was irradiated during whole development only (i.e., SC3).

### Sample preparation for dpMPK-1 assay and germ cell count

Following different exposure scenarios, wildtype N2 worms were all collected at the L4-YA (Young adult) stage, rinsed in M9 and quickly dissected and treated for dpMPK-1 (Sigma) staining according to the Gervaise and Arur, 2016 protocol [22]. The MT1522 strain, ~20 worms per condition (CTRL vs SC3) were picked in individual petri dishes for reproduction assay, while the rest were rinsed in M9 and treated for DAPI staining and sperm counting.

### Reproduction assay

Cumulated larvae number were quantified for each individual (20 per scenario) for eight days. Nematodes were transferred to a fresh NGM petri dish every 24 hours. In addition, newly laid eggs were quantified every 6 hours after the transfer every day.

### Spermatids quantification

Samples were washed three times with M9 and 15 to 25 worms per replicate were mounted on slides pre-coated with Poly-Lysine (10 μL at 0.1%). Worms were immobilized with 3 μL of 0.1 mM levamisole and dissected using a 0.45 μm gauge needle, then fixed with 2% paraformaldehyde. The freeze-cracking method was used to remove the cuticle [28]. The slides were then fixed in methanol/acetone (1:1) for 20 min at −20°C, then rinsed three times in 1x-PBST. The slides were stained with DAPI mounting medium for 2 hours at +4°C in a dark chamber. Images were obtained with an Axioobserver ZEISS Z1 microscope (Carl Zeiss AG, Oberkochen, Germany) equipped with a DAPI filter system, at 40× and 12-bit resolution and Z-stacked for each spermathecal. Spermatids were quantified using the FIJI (Fiji Is Just) ImageJ 2.1.0/1.53c software [29].

### Image acquisition and quantification of dpMPK-1 intensity and sperm number

For dpMPK-1-stained wildtype worms, images were collected on a Zeiss LSM780 confocal microscope (Carl Zeiss, https://www.zeiss.com/) at the Zone d’Observation en Microscopie" (ZoOM) platform at the "Commissariat à l’énergie atomique et aux énergies alternatives" (CEA, Cadarache, France) using a 40x magnification lens (Plan Apo water N.A. 1,2). Images were acquired in 12 bits using Zen black software (SP2 v.11.0, 2012, Carl Zeiss). Alexa Fluor 555 conjugated to anti-mouse secondary antibodies (InVitrogen, Waltham, MA, USA) was excited with 561nm Diode-Pumped Solid State (DPSS) lasers and emitted light was collected from 564 to 573nm using the MBS 561 filter. DAPI was excited with 405nm lasers and emitted light was collected from 450 to 495nm using the MBS 405 filter.

The expression of dpMPK-1 in the germline was measured as described by Gervaise & Arur, 2016 [22]. Important inter-individual variability of dpMPK-1 raw intensity was observed within conditions, especially for conditions with lower sample size (e.g., SC1) (S3 Fig). Therefore, to alleviate this variability, a reference zone in which no difference of intensity was observed between conditions was selected to weigh the raw intensity. No significant difference was observed between conditions in the loop area (S3 Fig), and intensities were similar to zone 2, therefore, the loop area was selected as the reference zone following the method of Eberhard et al [30]. Then, the ratio of intensity between the zone of interest and the reference zone was used to compare conditions as shown in S2 Fig. The intensity value used for statistical comparison is thus the ratio of the intensity at 80μm/0μm for zone 1, and -1 oocyte/0μm for zone 2.

Cell proliferation on DAPI stained gonads was measured using the spots tool on IMARIS software v.9.9 (Oxford Instruments, Abingdon, UK).

For MT1522 worms, images were acquired using ZEISS apotome as previously described [11]. Sperm number was manually counted using the ImageJ/FIJI software [29].

### Statistical analysis

All data was obtained with a simple random sampling. Count data (spermatids count, total brood size, ovulation rate, mitotic cell count) was analysed as functions of irradiation conditions (i.e., SC1, SC2, SC3) using a GLM (General Linear Model) following a quasi-Poisson or gaussian distribution. For statistical significance of intensity, the data was log-transformed to follow a normal distribution, means between conditions were compared using an Anova. For each test, post-hoc analysis was conducted (Dunnett for parametric tests) and p-values were adjusted using the Bonferroni correction. An alpha risk of 5% was taken as significant. Statistical analysis was conducted on R Studio software, Version 1.1.423 (© 2009–2018 RStudio, Inc., Boston, MA USA), using the following packages for statistical tests and data visualization: ‘ggplot2’, ‘ggpubr’, ‘dplyr’, ‘tidyr’, ‘car, ‘multcomp’.

## Results

We investigated two molecular pathways in reprotoxic response to chronic exposure to ionizing radiation at different life stages in *C*. *elegans*: Ras/ERK (MPK-1) pathway and apoptotic pathway through CED-3. S1 Fig shows the experimental design and different exposure scenarios seen in all the following figures.

### MPK-1 is activated in the pachytene zone of the germline upon chronic exposure to IR

**Fig 1:**
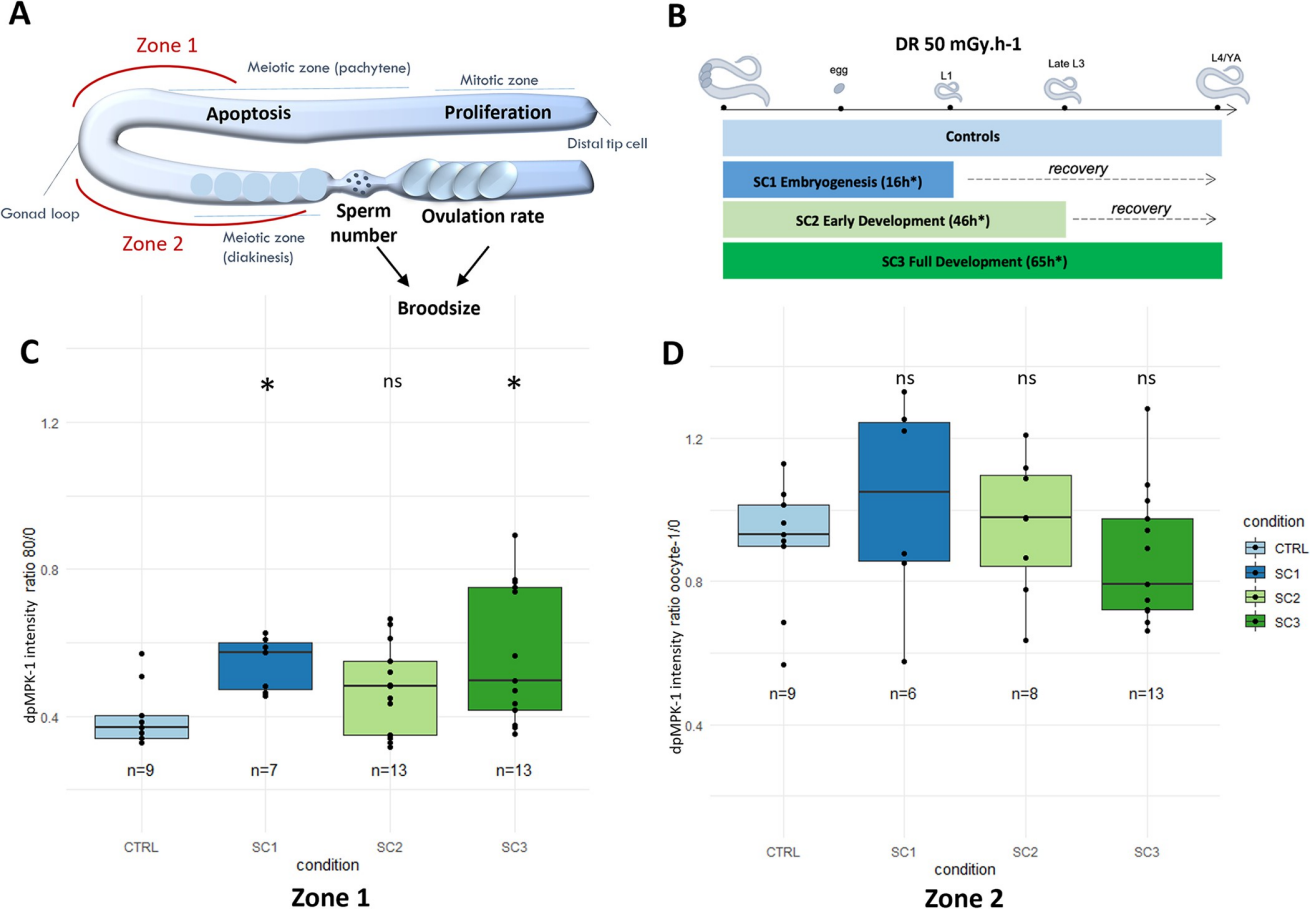
Analysis of dpMPK-1 spatial expression in N2 *C*. *elegans* hermaphrodite germline after chronic exposure to IR at different life-stages. (A) dpMPK-1 is expressed in two distinct zones of *C*. *elegans* hermaphrodite gonad. In zone 1, germ cells are in the pachytene stage of the meiosis where apoptosis occurs. In zone 2, germ cells are maturing into oocytes and ovulation occurs. (B) N2 worms were exposed chronically at a fixed dose rate (DR) of 50 mGy.h^-1^ over different developmental periods. All scenario of exposure started from *in utero* exposure until–L1 stage (SC1)–Late L3 stage (SC2)–L4/YA stage (SC3). (C) dpMPK-1 intensity in Zone 1 for each condition (B) dpMPK-1 intensity in Zone 2 for each condition (Anova, Dunnett post hoc with Bonferroni adjustment, ‘*’ p-value < 0.05, ‘ns’ non-significant).

**Fig 2:**
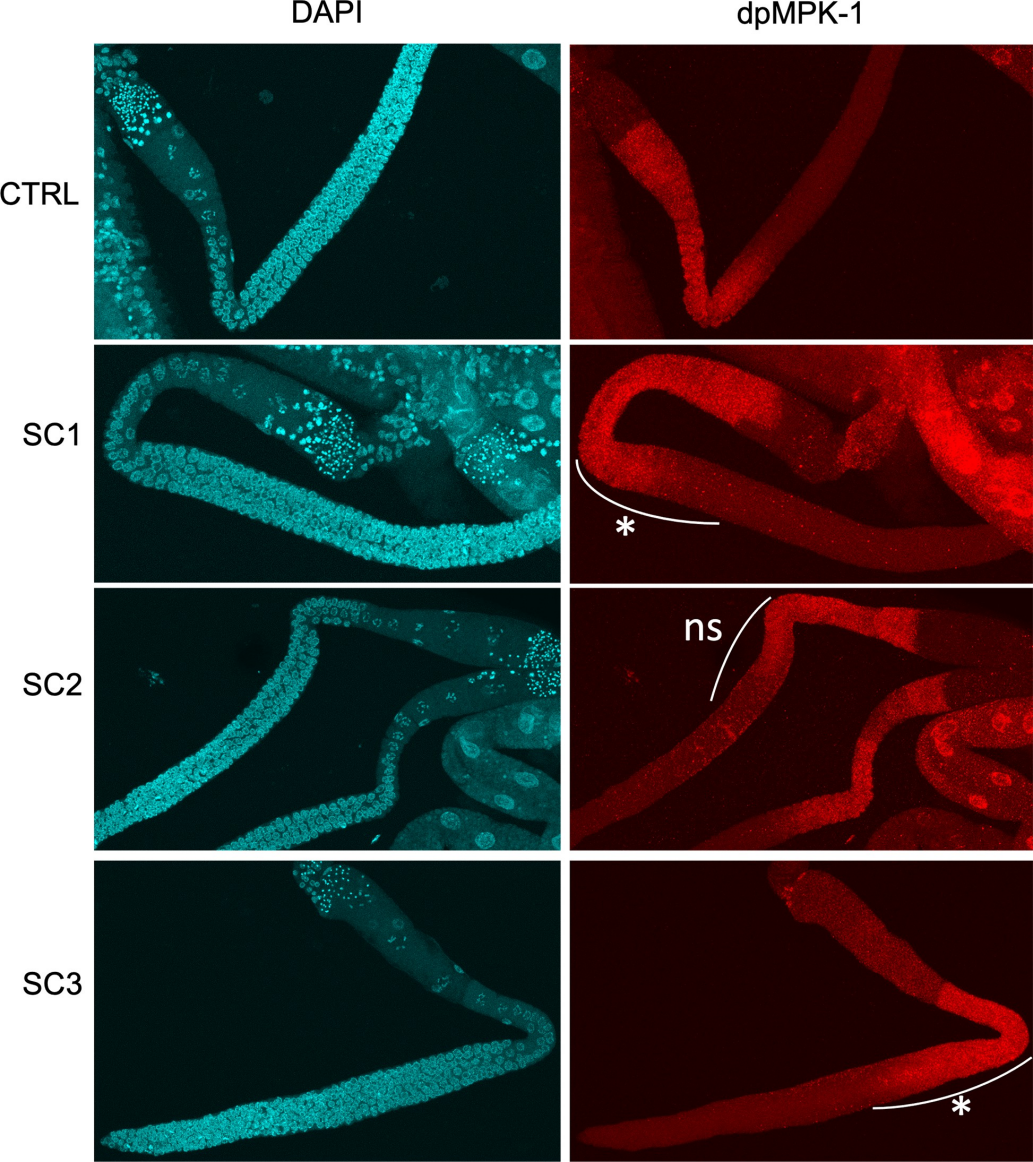
MPK-1 phosphorylation is induced in early pachytene (Zone 1) in wild-type germlines following irradiation at different life stages. (SC1: *in utero* until embryogenesis; SC2: *in utero* until the beginning of gametogenesis; SC3: *in utero* until L4/YA–whole development–see S1 Fig) ‘*’ p-value < 0.05; ‘ns’ non-significant.

Upon exposure to many stressors, including IR, MPK-1 is activated, i.e., double-phosphorylated (dpMPK-1). Here, we investigated dpMPK-1 expression in the germline in different zones of the germline that reflect different mechanisms that could occur upon exposure at different life stages (i.e., cell proliferation, progression into meiosis, germ cell apoptosis in Zone 1, and ovulation and maturation of oocytes in Zone 2) as shown in Fig 1A. S2 Fig shows the method for intensity measurement of dpMPK-1 in *C*. *elegans* hermaphrodite germline. Fig 2 shows confocal microscopy images of dpMPK-1 and DAPI stained *C*. *elegans* L4/YA (young adult) hermaphrodite germlines after each exposure scenario.

dpMPK-1 expression in *C*. *elegans* germline is spatially and temporally dynamic and controls various processes linked to reproduction as shown in Fig 1A. As dpMPK-1 expression is tightly linked to germline development across larval stages, worms were irradiated, and dpMPK-1 expression assessed at different developmental periods as shown in Fig 1B. In zone 1, dpMPK-1 is significantly over expressed at 80μm from the loop after irradiation throughout the whole development (SC3) and after irradiation throughout embryogenesis (SC1) as shown in Figs 1C and 2. Meanwhile, no difference in intensity between conditions was observed at 40μm from the reference zone (S3 Fig). This can suggest a precocious activation of MPK-1 in the germline after irradiation, possibly affecting cell proliferation and meiotic progression, and later germ cell apoptosis. To validate this hypothesis, we analysed (i) the cell number and density in the mitotic zone of the gonad, and (ii) reproduction decrease in the non-apoptotic *ced-3(n717)* mutant. In both cases, a focus was made on the SC3 condition which showed the highest increase of dpMPK-1 staining.

On the other hand, in zone 2, where dpMPK-1 controls the oocyte-linked processes (maturation, ovulation) [22], no significant difference between irradiated conditions and control was found (Fig 1D), concordant with the hypothesis that a deficit in oogenesis occurs earlier during oocyte development.

### Total cell number and density in the mitotic zone of the gonad is unchanged upon chronic exposure to IR

**Fig 3:**
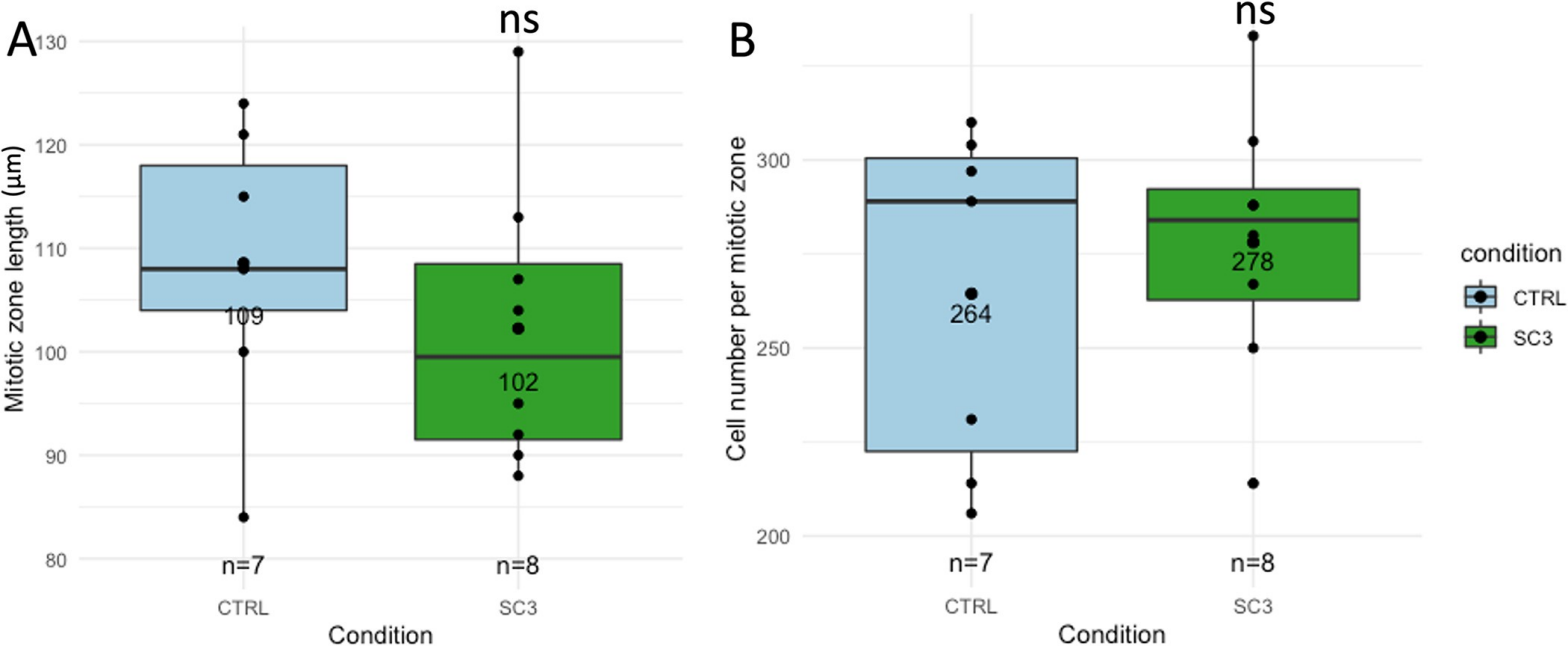
Analysis of cell proliferation in the mitotic zone of N2 *C*. *elegans* hermaphrodite germline. (A) Mitotic zone length for each condition (CTRL = 109±13 μm, SC3 = 102±14 μm, GLM, Dunnett contrasts, p-value = 0.37) (B) Cell number per mitotic zone (CTRL = 264±45, SC3 = 278±36, GLM, Dunnett contrasts, p-value = 0.51), ‘ns’ non-significant.

Because MPK-1 was activated in the pachytene region of the germline, where it controls germ cell proliferation, we investigated whether or not germ cell number and density was affected after chronic exposure to IR. Methodology for cell proliferation analysis in the mitotic zone is shown in S4 Fig. Fig 3A shows the mitotic zone length for each condition, while Fig 3B shows total number of cells per mitotic zone for each condition.

A slight decrease of mitotic zone length can be observed in irradiated worms (Fig 3A; mean±SD for CTRL = 109±13 μm, SC3 = 102±14 μm, GLM, Dunnett contrasts, p-value = 0.37), however non-significant. Similarly, no difference was observed in the total number of cells in irradiated worms compared to controls (Fig 3B; mean±SD for CTRL: 264±45, SC3: 278±36, GLM, Dunnett contrasts, p-value = 0.51). Therefore, it seems that IR has no effect on cell proliferation in the mitotic zone of the germline under our conditions.

### Apoptotic pathway induces ovulation rate decrease but no effect on sperm upon chronic exposure to IR

**Fig 4:**
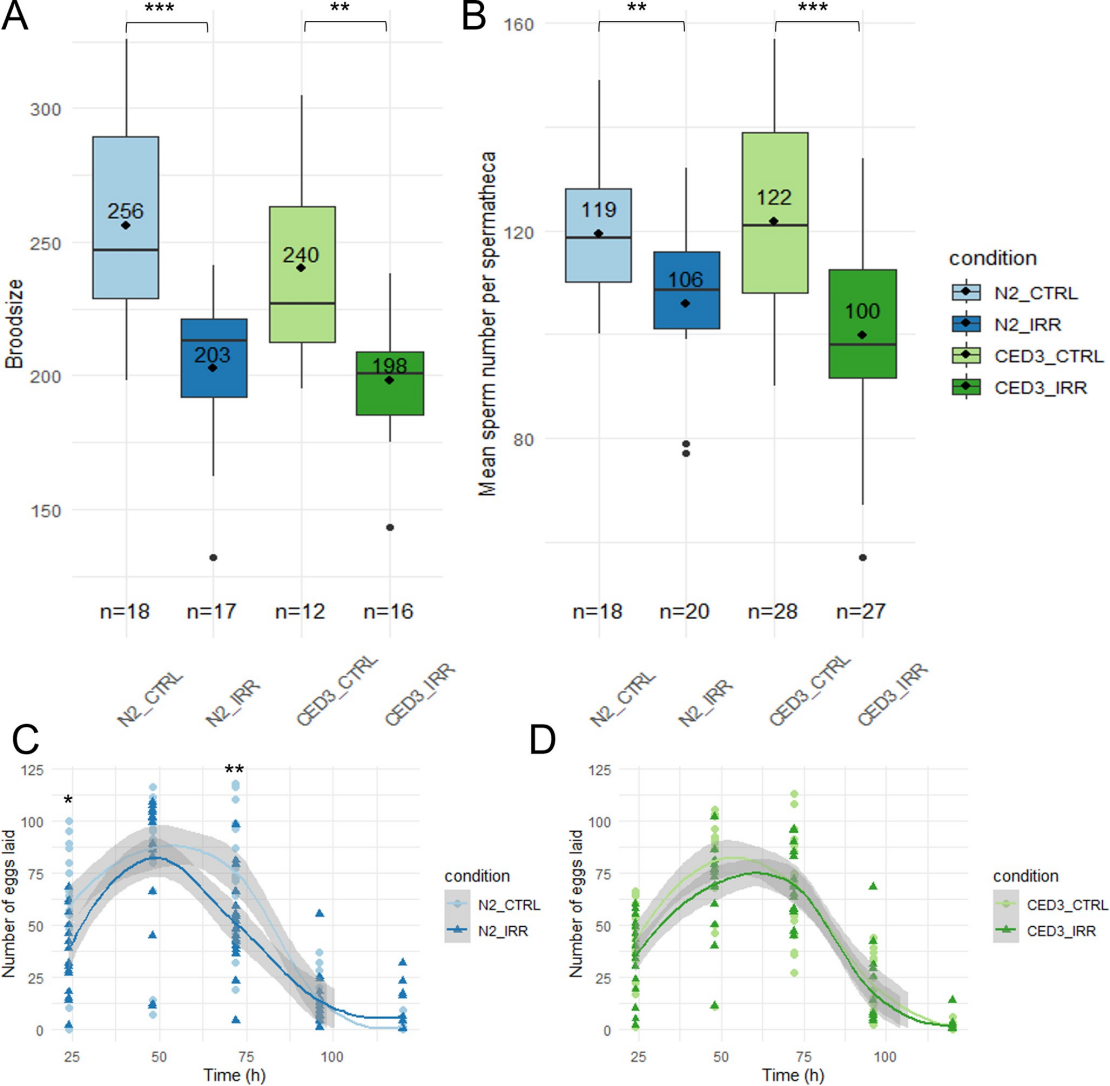
Analysis of radio-induced reprotoxic effects in non-apoptotic *ced-3(717)* mutant worms vs N2 worms. *ced-3(n717)* vs N2 analysis of (A) brood size, (B) sperm count and (C, D) ovulation rate in CTRL vs SC3 (irradiated during whole development) (GLM, Tukey post-hoc; ‘*’ p value < 0.05, ‘**’ p value < 0.01, ‘***’ p value < 0.001).

Because MPK-1 was activated in the pachytene region of the germline where it controls apoptotic pathway, we investigated whether apoptosis was induced after chronic exposure to IR. Apoptotic loss of function *ced-3(n717)* worms were analysed under the same conditions as wildtype N2 worms to understand the effects of apoptosis in the gamete response after irradiation. Fig 4 shows the total brood size (A), sperm count (B) and ovulation rate (C, D) of *ced-3(n717)* and N2 worms.

Brood size decrease is observed in both strains (N2 and *ced-3(n717)*) upon chronic exposure to IR throughout the whole development (Fig 4A, -21% with p-value < 0.001 and -17% with p-value = 0.005 respectively). Sperm number is reduced in both irradiated *ced-3(n717)* worms and N2 worms compared to controls (Fig 4B, -18% with p-value < 0.001 and -11% with p-value = 0.004). Interestingly, *ced-3(n717)* worms show a similar decrease in brood size and sperm number while N2 worms show a bigger decrease in brood size than in sperm number. Thus, we conclude that in N2, sperm count is not reduced via apoptosis, and that brood size decrease can be partly attributed to another pathway than sperm decrease. We hypothesize that this decrease can be attributed to oocyte apoptosis.

The effect of apoptosis on ovulation rate was measured by counting the number of eggs laid per hour (fertilized or not, thus independently from sperm number). In N2 worms, there is a significant decrease in ovulation rate of irradiated worms compared to controls (Fig 4C, GLM, Tukey contrasts, p value = 0.015 at 24h, p value = 0.008 at 72h). Interestingly, in the *ced-3(n717)* strain, there is no significant difference in ovulation rate between irradiated and control worms (Fig 4D). Thus, we conclude that in irradiated N2, ovulation rate is decreased via oocyte apoptosis, thus contributing to the total brood size decrease via another pathway than sperm decrease.

## Discussion

### MPK-1 activation in the pachytene region reveals contribution of apoptosis rather than cell proliferation defects

Our study aimed to decipher the underlying mechanisms at different biological scales of “brood size decrease” adverse outcome, which is crucial for population dynamics. Based on results from our previous studies [10–12,15,26,31], we focused on processes involved in germline development and stress response such as the Ras/ERK (MPK-1) pathway and apoptotic core machinery. As previously described, MPK-1 is involved in many processes such as the regulation of sperm count [19,32], germ cell proliferation [33–35] and differentiation [36–39], but also in stress response pathways [20,21,26,40]. No significant difference in dpMPK1 expression was observed in the ‘ovulation zone’ (i.e., zone 2), meaning that maturation of oocytes and ovulation processes are not affected by IR under our exposure conditions. Thus, the 10% ovulation rate decrease that was previously observed [11] might be the consequence of prior effects occurring during oocyte proliferation and differentiation. Similarly, we observed no significant difference of dpMPK-1 expression in the loop area (cells transitioning from late pachytene to early diplotene). On the contrary, our results show an overexpression of dpMPK-1 upon exposure throughout the whole development in the early to late pachytene zone (i.e., zone 1), where it controls the progression of cells into meiosis, and apoptosis as previously described [21,30]. In addition, it is also concordant with the overexpression of *egl-1* observed in similar conditions since its activation is required for IR–induced *egl-1* transcription [15,21]. MPK-1 activation in this zone can be involved in several processes, notably sperm-promotion and cell proliferation [41–44]. Under our conditions, it is not linked to sperm promotion since a decrease of sperm count was observed. In addition, germ cell proliferation investigation in the mitotic zone did not show any significant result neither on mitotic zone length nor on germ cell density under our conditions, with a mean number concordant with the literature (i.e., ~236 mitotic cells) [45]. To conclude, the Ras/ERK (MPK-1) pathway is activated in the pachytene region of the gonad in *C*. *elegans* upon chronic exposure to IR. This activation does not lead to any observable effects on germ cell proliferation but could trigger the apoptotic pathway and lead to gamete decrease.

### Apoptosis is responsible for oocyte decrease but not spermatocyte decrease

Previous studies had shown the induction of apoptosis in *C*. *elegans* via several pathways upon chronic exposure to IR. Notably, the expression of the proapoptotic gene *egl-1* [15] was increased as well as the presence of apoptotic cell corpses [10,23], and differential regulation of miRNA family *mir-35*, also involved in fecundity and embryonic development [12,44]. Despite this upstream evidence, it was not clearly known to what extent the apoptotic pathway contributes to downstream brood size decrease. Our analysis of the core apoptotic pathway revealed that radio induced apoptosis is not involved in sperm decrease but is involved in ovulation rate decrease. Indeed, sperm number is decreased after irradiation in *ced-3(n717)* mutants in the same way as in N2 [11], which means that this decrease occurs via a mechanism other than apoptosis. This result is concordant with the literature, where acute exposure to IR did not induce apoptotic machinery in male germ cells [24–26]. In addition, this is similar to what is generally observed in invertebrates, i.e., that sperm has poor DNA repair capacities and limited antioxidant defences [46]. Thus, sperm decrease must be due to another, yet unknown mechanism. On the other hand, ovulation rate is not affected by irradiation in *ced-3(n717)* mutants, contrary to what was observed in N2, which means apoptosis is involved in the decrease of the ovulation rate in N2 after chronic irradiation, probably even independently from the regulation by spermatozoa, notably via the Major Sperm Protein [18,47]. This result is also concordant with physiological germline apoptosis already occurring during normal oogenesis increased with ionizing radiation (as *egl-1* induction has previously been observed in our conditions).) [15,48]. In conclusion, brood size decrease in *ced-3(n717)* mutants can be attributed to sperm decrease only. This confirms that brood size decrease in wildtypes is not only due to sperm decrease but also to ovulation decrease, via the apoptotic pathway.

**Fig 5:**
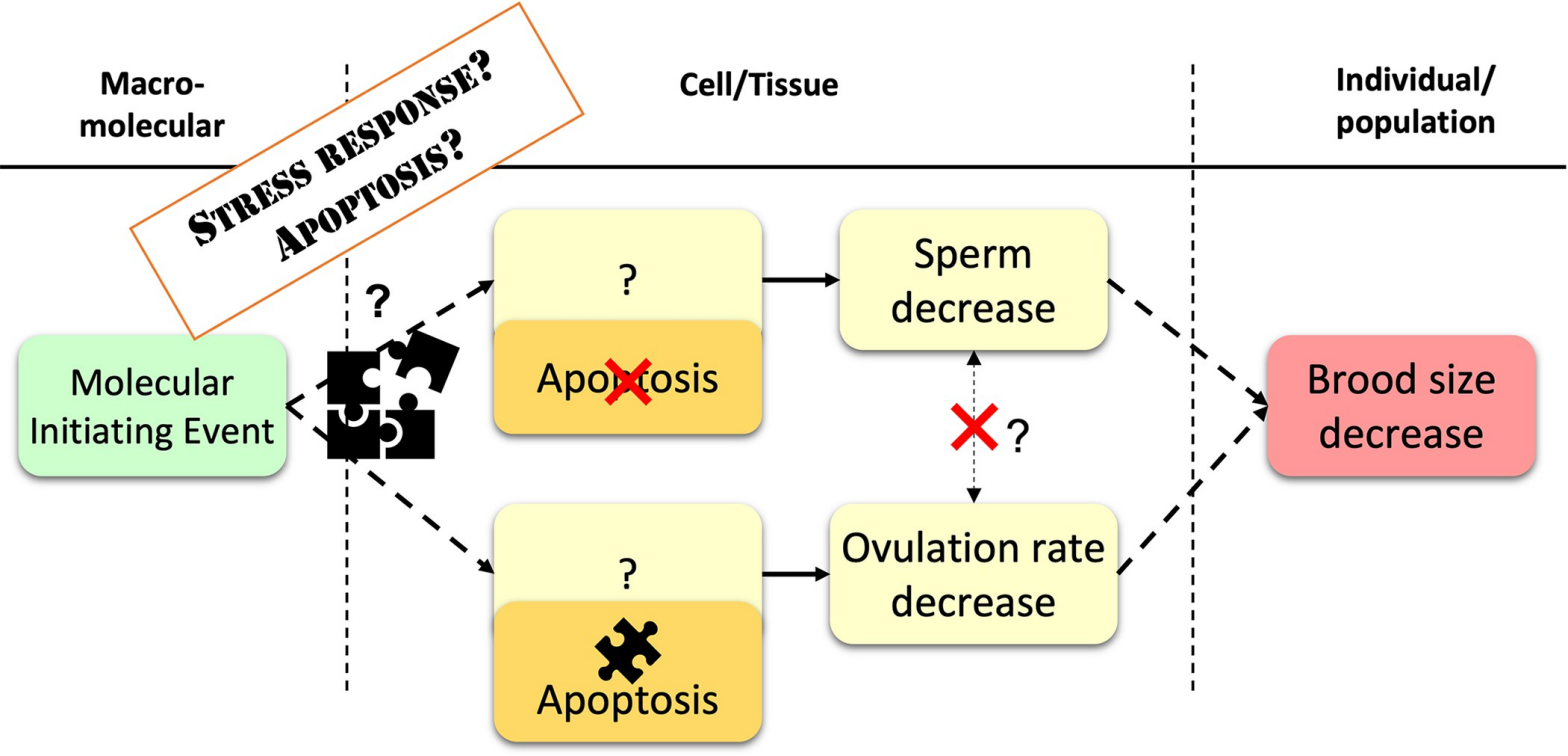
Proposed steps leading to brood size decrease Adverse Outcome in hermaphrodite *C*. *elegans* exposed to chronic ionizing radiations. Two mechanisms act in parallel that affect oogenesis and spermatogenesis independently and lead to brood size decrease. Oocytes are reduced directly and only via apoptosis, while spermatocytes are reduced via another yet unknown mechanism. Question marks represent missing key events or key event relationships. The puzzle symbol represents key events that were validated in this study. The red cross represents key events and key event relationships that were invalidated in this study.

From our study, we suggest that two mechanisms act in parallel that affect spermatogenesis and oogenesis independently, both contributing to brood size decrease (Fig 5). The causal mechanisms of sperm decrease remain unknown, but we show that neither the Ras/ERK (MPK-1) pathway nor apoptotic pathway are involved in this decrease.

### Causes of sperm decrease: Perspectives from our results and the literature

In previous studies, sperm count was shown to be decreased only after exposure throughout the whole development, and not only in precocious stages [11] (with the same dose rate) nor throughout gametogenesis ([10] with an equivalent total dose). These results suggest that there is cumulative damage during larval development, culminating in sperm decrease in adulthood. In many species, sperm decrease is mediated via DNA damage-induced apoptosis [49–51]. In this study, it was shown that apoptosis is not involved in sperm decrease in *C*. *elegans*. Similarly, no effect was observed either in the length or the cell density of the mitotic zone, showing that sperm count decrease is apparently not linked to germ cell proliferation defect. However, several previous studies on *C*. *elegans* identified a potential slowdown of germ cell proliferation showing an increase in cell cycle arrest in the mitotic zone of the gonad after acute exposure to IR [26], or chronic exposure from 37 mGy.h^-1^ [15] or a decrease in expression of proteins linked to the MCM complex involved in DNA replication and critical for cell division [14]. Therefore, a decrease in germ cells proliferation speed probably due to DNA damage and repair could directly induce a decrease in the number of spermatozoa since they are produced on a finite time-lapse. Considering all these lines of evidence, this hypothesis cannot be discarded and needs to be further investigated to fill the knowledge gap and finally identify the missing key event of brood size decrease in *C*. *elegans*.

## Conclusion

In line with our previous studies showing a decrease of hermaphrodite *C*. *elegans* reproduction upon chronic exposure to IR, this study aimed to investigate more specific mechanisms linked to gamete development and stress response. We showed effects in both oocytes and spermatocytes but via different mechanisms. Indeed, oocytes seemed to be only affected by apoptosis via the Ras/ERK (MPK-1) pathway which led to a slight decrease in ovulation rate. On the other hand, spermatocytes were not affected by apoptosis, but we still observed a reduction in sperm count that can be hypothetically attributed to a slowdown in proliferation in germ stem cells. Thus, we show that reprotoxic response to chronic IR involves germ cell sex-specific mechanisms. Interestingly, this work can contribute to understanding why, after multigeneration exposure, it is observed that individual brood size still decreases continuously over time, despite an increase in males in the population to rescue fecundity [13]. This continuous decrease could be partly explained by the 10% oogenesis decrease due to apoptosis throughout adulthood. Thus, only looking at the individual scale could make it possible to think that sperm decrease is critical to reproduction decrease (90%) contrary to oocytes (10%). However, on a larger scale, sperm decrease is rescued by the increase in males in populations, while oocytes keep decreasing and become mainly responsible for reproduction decrease at the population level. This result can be linked to effects at larger biological scales, giving a meaningful interpretation for ecological risk assessment. Further research is needed to determine the mechanisms of sperm decrease, possibly via cell proliferation defects and to understand the causes of radiosensitivity of these gametes in *C*. *elegans* and potentially other organisms.

## Supporting information

S1 Fig Experimental design for analysis of life stage dependent reprotoxic effects (adapted from Dufourcq-Sekatcheff *et al* 2021). (SC: scenario, OP50: E. Coli strain, L1-L4: C. elegans larval stages, YA: Young Adult).
(TIF)

S2 Fig Method for measuring dpMPK-1 expression in *C*. *elegans* germline. (A) DAPI-stained L4/YA hermaphrodite *C*. *elegans* germline. Germ cells differentiate from the distal end of the gonad (distal tip cell on the left) to the proximal end (spermatheca on the right). (B) dpMPK-1 expression in *C*. *elegans* dissected gonad (zone 1 corresponds to expression in the pachytene stage, 2 measures of intensity were taken at 80μm and 40μm from the reference zone (0μm); zone 2 corresponds to expression in the most proximal oocyte).
(TIF)

S3 Fig Raw intensity of dpMPK-1 in different areas of the germline for each condition of exposure. (A) Intensity in Zone 1 at 80μm from the loop area towards the distal end of the gonad (B) Intensity in Zone 1 at 40μm from the loop area towards the distal end of the gonad (C) Intensity in the loop area (D) Intensity in Zone 2 (-1 oocyte). (Kruskal Wallis, Dunn test with Holm adjustment, ‘*’ p-value < 0.05, ‘ns’ non-significant).
(TIF)

S4 Fig Automated cell counting of mitotic cells in the *C*. *elegans* distal gonad. Mitotic Zone extends from Distal Tip Cell to first meiotic cells. Left: 3D z-stack of a DAPI-stained dissected gonad, right: Automated spot counting of the same gonad using IMARIS software.
(TIF)

S1 Data (XLSX)
