# Supplementary figures and images for: Two distinct mechanisms lead to either oocyte or spermatocyte decrease in C. elegans after whole developmental exposure to γ-rays
Source: PLoS One. 2023 Nov 27;18(11):e0294766. doi: 10.1371/journal.pone.0294766 (PMC10681227; doi:10.1371/journal.pone.0294766)

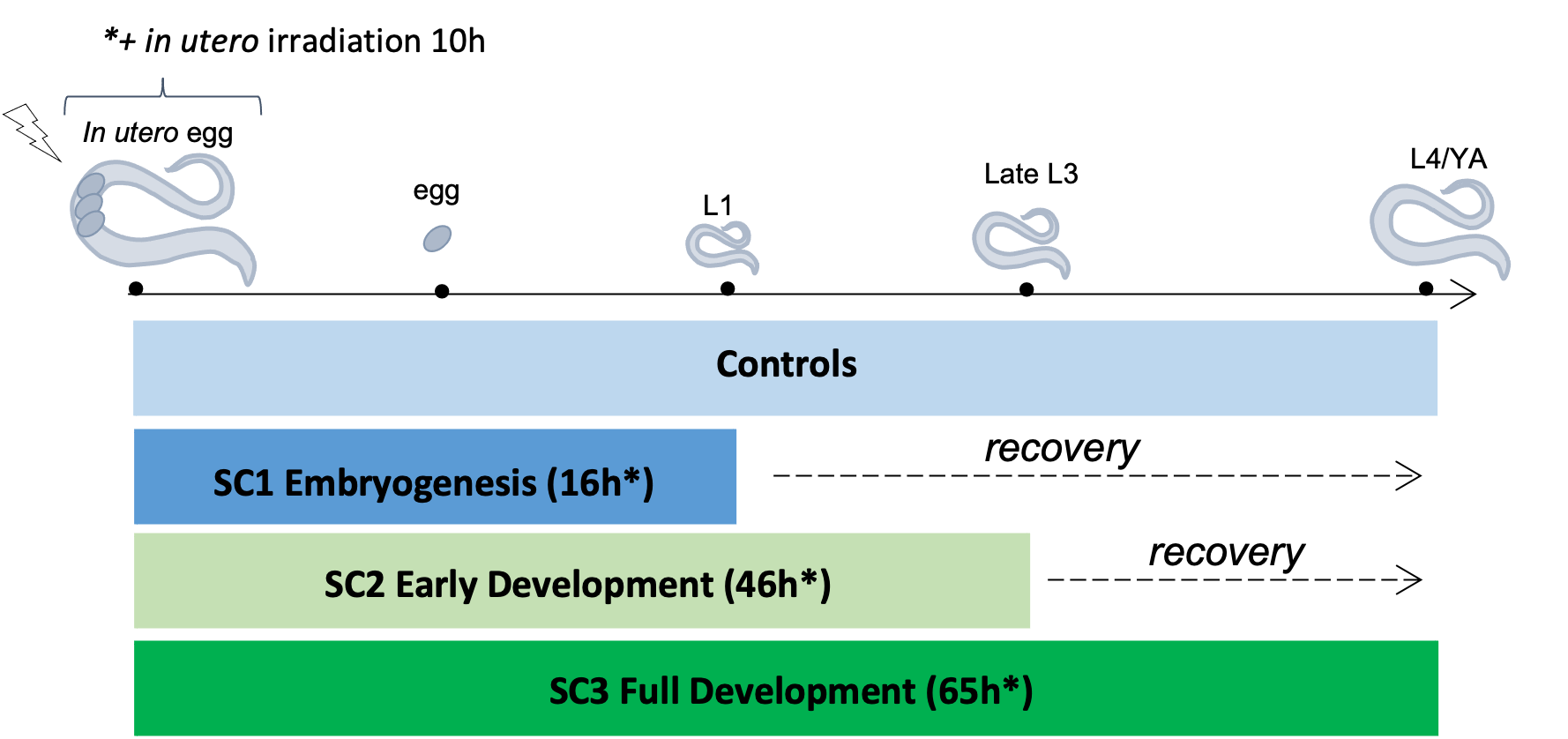

Supplement: S1 Fig — (SC: scenario, OP50: E. Coli strain, L1-L4: C. elegans larval stages, YA: Young Adult). (TIF) [file pone.0294766.s001.tif]

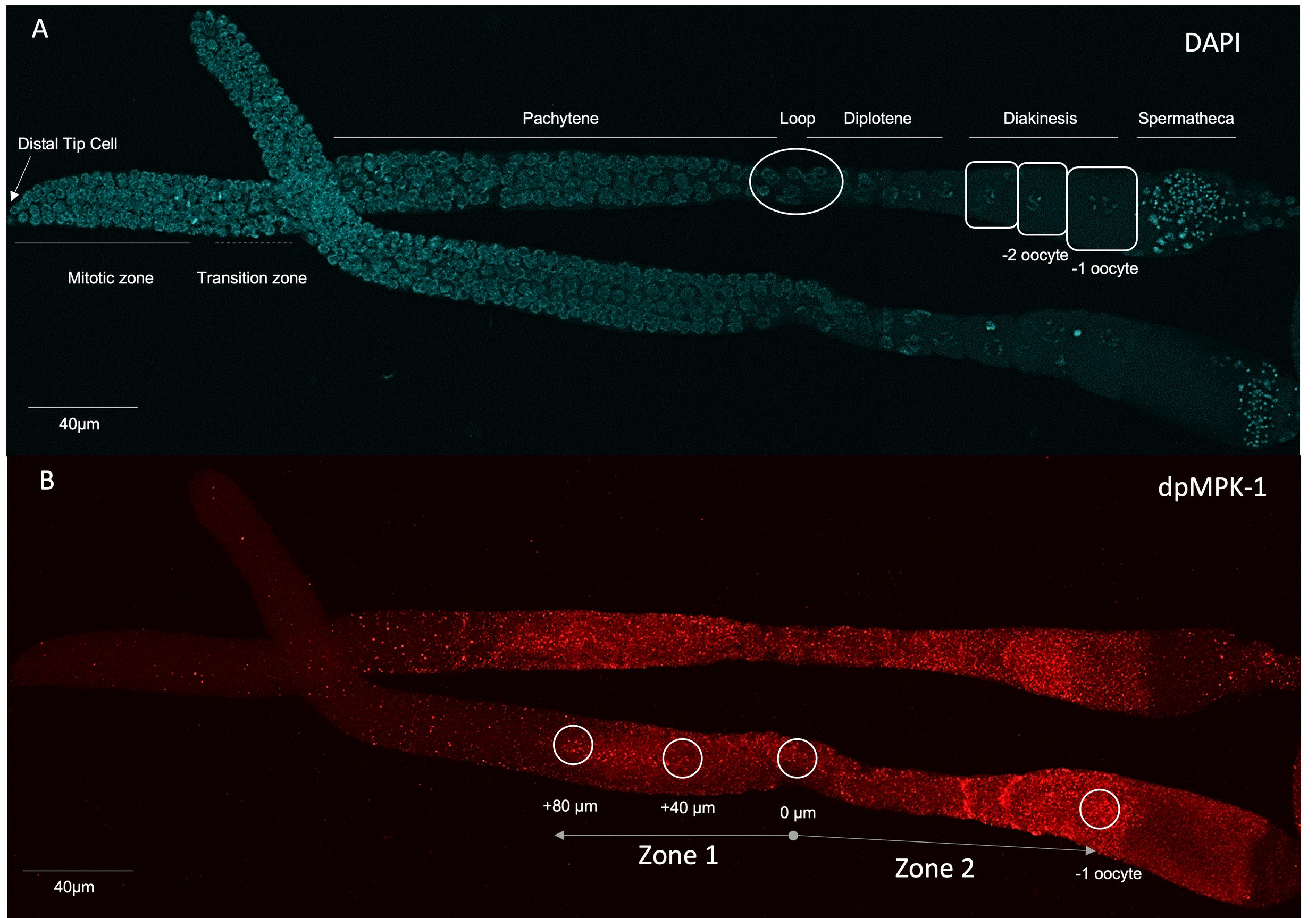

Supplement: S2 Fig — (A) DAPI-stained L4/YA hermaphrodite C. elegans germline. Germ cells differentiate from the distal end of the gonad (distal tip cell on the left) to the proximal end (spermatheca on the right). (B) dpMPK-1 expression in C. elegans dissected gonad (zone 1 corresponds to expression in the pachytene stage, 2 measures of intensity were taken at 80μm and 40μm from the reference zone (0μm); zone 2 corresponds to expression in the most proximal oocyte). (TIF) [file pone.0294766.s002.tif]

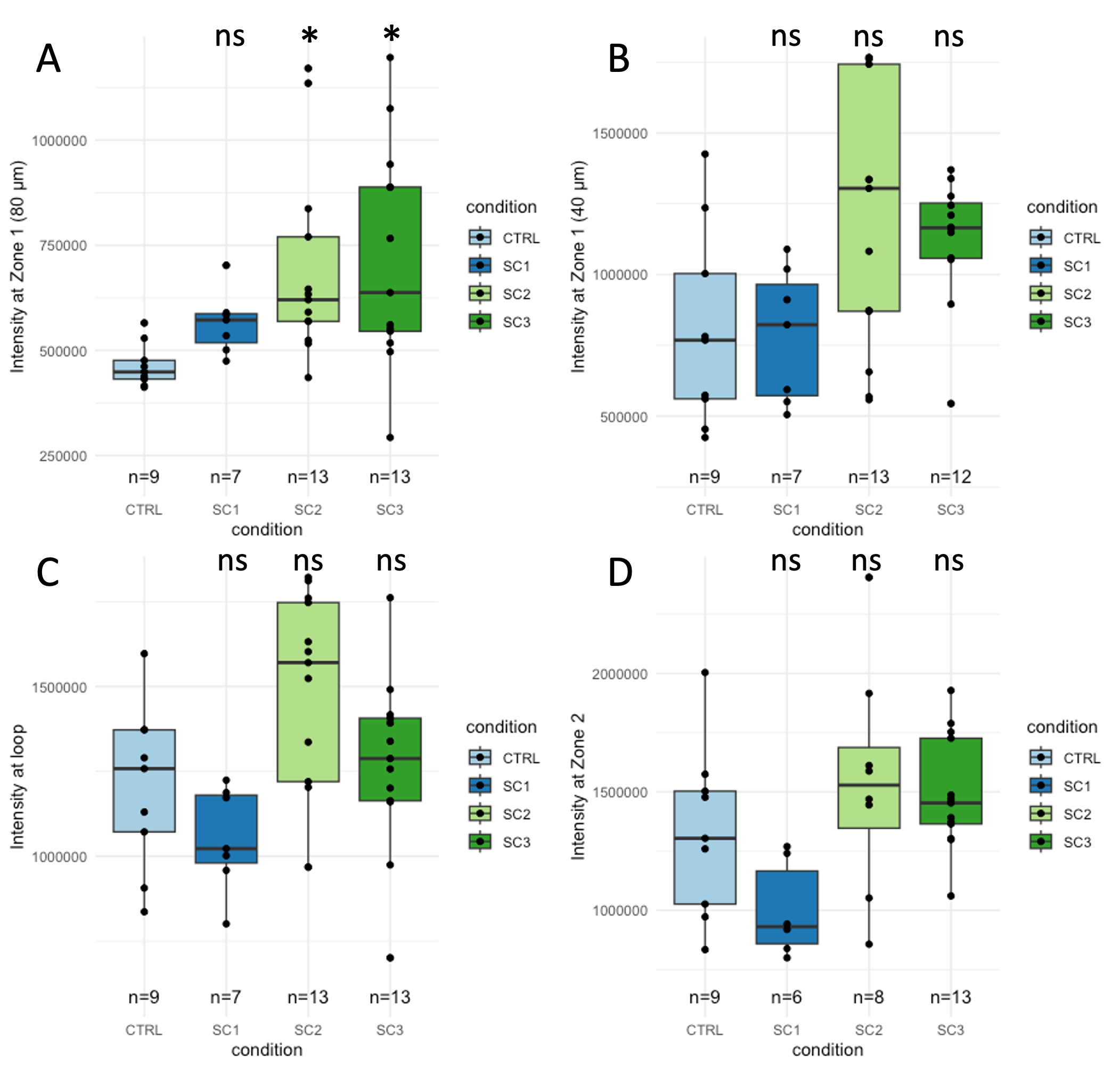

Supplement: S3 Fig — (A) Intensity in Zone 1 at 80μm from the loop area towards the distal end of the gonad (B) Intensity in Zone 1 at 40μm from the loop area towards the distal end of the gonad (C) Intensity in the loop area (D) Intensity in Zone 2 (-1 oocyte). (Kruskal Wallis, Dunn test with Holm adjustment, ‘*’ p-value < 0.05, ‘ns’ non-significant). (TIF) [file pone.0294766.s003.tif]

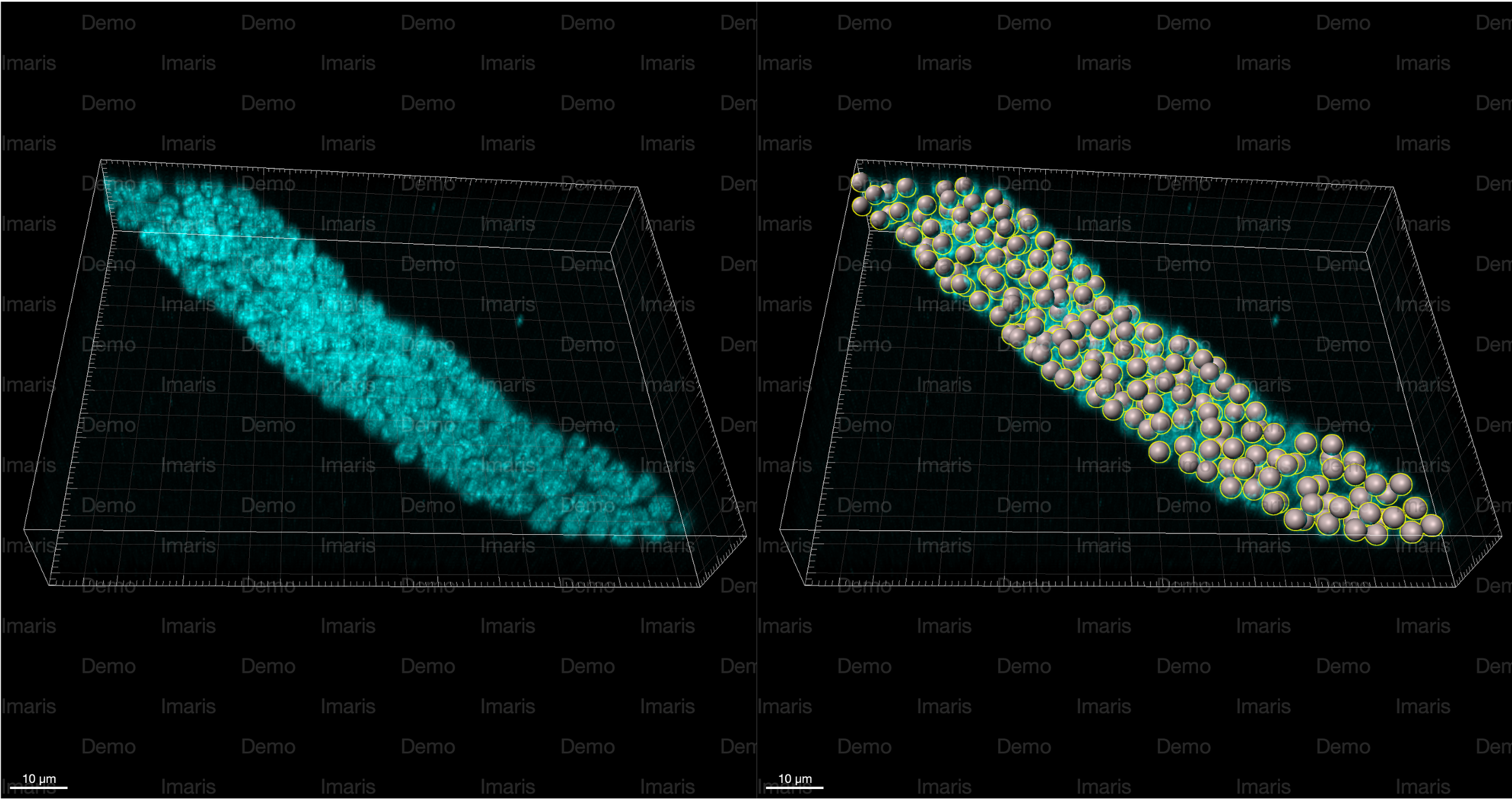

Supplement: S4 Fig — Mitotic Zone extends from Distal Tip Cell to first meiotic cells. Left: 3D z-stack of a DAPI-stained dissected gonad, right: Automated spot counting of the same gonad using IMARIS software. (TIF) [file pone.0294766.s004.tif]
